# The Prevalence of Personality Disorders among Emergency Nurses Based on MMPI-2 Questionnaire; a Cross-sectional Study

**Published:** 2017-01-09

**Authors:** Parvin Kashani, Sahar Mirbaha, Mohammad Mehdi Forouzanfar, Farahnaz Meschi, Alireza Baratloo

**Affiliations:** 1 Department of Emergency Medicine, Loghmane Hakim Hospital, Shahid Beheshti University of Medical Sciences, Tehran, Iran.; 2 Department of Emergency Medicine, Shohadaye Tajrish Hospital, Shahid Beheshti University of Medical Sciences, Tehran, Iran.; 3 Department of Clinical Psychology, Karaj Branch Islamic Azad University, Alborz, Iran.

**Keywords:** Personality disorders, burnout, professional, workplace, nurses, emergency department

## Abstract

**Introduction:**

The prevalence of behavioral disorders is substantially higher in stressful working environments such as emergency departments. The present study aimed to evaluate the prevalence of personality disorders among emergency nurses.

**Methods:**

In the present epidemiologic study, the prevalence of personality disorders among emergency nurses of three educational hospitals, Tehran, Iran, were evaluated based on Minnesota Multiphasic Personality Inventory-2 (MMPI-2) test. After the questionnaires were filled, data were entered to a special software for MMPI-2 test and the final result was interpreted based on the opinion of a clinical psychologist. Findings were reported using descriptive statistics.

**Results:**

102 emergency nurses with the mean age of 30.2 ± 5.6 years were enrolled (100% female; 100% with master’s degree in nursing). The mean working time and experience of studied nurses were 210.8 ± 47.9 hours/month (130-370) and 4.1 ± 3.6 years (1-20), respectively. 32 (31.4%) cases showed symptoms of personality disorders The most common personality disorder detected in this study was somatization with 8.8%, hysteria with 6.9% prevalence, and pollyannaish with 4.9%. Among the studied factors only recent history of unpleasant event has significant correlation with existence of personality disorders (p = 0.015).

**Conclusion:**

The present study showed that somatization, hysteria, and pollyannaish were the most common personality disorders among the emergency nurses. History of an unpleasant event in the past year was the only effective factor in existence of personality disorders in the studied nurses.

## Introduction

Workplace stress is one of the most important causes of mental illnesses as statistics show that one fourth of the employed population have experienced some kind of job-related behavioral disorder (1). The prevalence of these behavioral disorders is substantially higher in stressful working environments such as emergency departments (ED). The medical staff, especially nurses and physicians, are at risk of physical and mental damage in ED due to the nature of their work, which involves high work load, long working shifts with insufficient number of personnel, lack of social support, lack of free time for resting, and encountering serious injuries, wounds and adverse scenes (2). A systematic review in the past year has shown the 26-35% prevalence of personality and mental disorders among ED nurses due to their job (3, 4). These disorders not only affect the personnel’s health, but also decrease their efficiency and the quality of services they provide (5). Meanwhile, the proper performance of hospital department personnel plays a major role in decreasing the burden caused by accidents and diseases, and increasing patient satisfaction (6, 7). 

Existing studies have demonstrated the effect of job burnout on prevalence of behavioral disorders. However, findings reveal that in the same workplace environment, people are not affected by job burnout and personality disorders equally. In other words, personal, family-related and social factors all exert an effect on the prevalence of these disorders (8, 9). Therefore, personality disorder is a multi-factorial condition, in which workplace stress, job burnout, and personal and social factors should be studied simultaneously. Although numerous studies are available in the field of personality disorders caused by workplace stress, scarcity of such information in Iran reveals the need for a study in this field. Therefore, the present study was designed aiming to evaluate the prevalence of personality disorders among emergency nurses.

## Methods

***Study design and setting***

The present study is a cross-sectional one, in which nurses working in emergency departments of 3 hospitals, Shohadaye Tajrish, Imam Hossein and Loghmane Hakim, Tehran, Iran, were questioned in 2015. Before asking the questions, written informed consent was obtained from all the nurses. The researchers adhered to the principles of Helsinki Declaration throughout the study.

***Participants***

The study population consisted of emergency nurses of the three mentioned educational hospitals. Not giving consent and not filling out the questionnaire correctly and the questionnaire being invalid based on Minnesota Multiphasic Personality Inventory-2 (MMPI-2) test were considered as exclusion criteria. Nurses were selected for questioning using simple random sampling. A list of nurses working at the hospitals was prepared for this purpose and participants were selected randomly.

***Data gathering***

Personality disorders were evaluated based on the standard MMPI-2 test. MMPI-2 is a standard questionnaire for gathering a wide range of self-described characteristics and scoring them, which is a quantitative index of the individual’s emotional adaptability and shows their attitude toward taking part in the test (10). MMPI test is the most famous and widely used personality questionnaire that has been developed as an objective tool for diagnosis of mental diseases. This test is a self-evaluation questionnaire with “yes” or “no” answers and has 3 validity scales and 10 clinical scales. Validity scales provide information regarding the subject’s approach to the test, while the 10 primary clinical scales are used for diagnosis of mental disorders. The most valuable use of MMPI-2 is in screening abnormal people generally and determining the severity of the problem specifically (11). Diagnostic layers and scales of MMPI-2 include hypochondriasis, depression, hysteria, psychopathic deviate, masculinity/femininity, paranoia, psychasthenia, schizophrenia, hypomania, and social introversion. To increase the clinical benefit of MMPI, 3 validity scales are present including lie detection scale, infrequency, and defensiveness as correction or inhibition scale. Demographic data and MMPI-2 test were included in a questionnaire, which was given to the studied nurses. 

***Definitions***

Recent unpleasant event was defined as any shocking tragic event during the previous year and recent trauma history was defined as any motor vehicle collision during the last year.

***Statistical analyses***

After the questionnaires were filled, data were entered to a special software for MMPI-2 test and the final result was interpreted based on the opinion of a clinical psychologist. Data were analyzed via STATA 11.0 statistical software and presented as mean ± standard deviation or frequency and percentage.

## Results

104 emergency nurses were questioned, 2 cases were excluded due to unreliable questionnaires. Finally, 102 participants with the mean age of 30.2 ± 5.6 years (23-49) were enrolled for analysis (100% female; 100% with master’s degree in nursing). 1 (1%) case had positive family history of known psychiatric disease. Table 1 presents baseline characteristics of the studied cases. 52.9% of cases were married, 59.8% had stable employment status, 82.4% had variable work shifts, 71.4% earned ≥ 500 US dollars each month, 85.3% did not have any medical history, 86.3% did not have drug history, 33.3% had history of recent unpleasant event, and 4.9% had recent history of trauma. The mean working time and experience of the studied nurses were 210.8 ± 47.9 hours/month (130-370) and 4.1 ± 3.6 years (1-20), respectively.

32 (31.4%) cases showed symptoms of personality disorders based on MMPI-2 interpretations. Table 2 shows the frequency of personality disorders among studied participants. The most common personality disorders detected in this study were somatization with 8.8%, hysteria with 6.9% prevalence, and pollyannaish with 4.9%.

Table 3 shows the relationship of demographic and baseline variables with personality disorders. Among the mentioned factors, only recent history of unpleasant event had significant correlation with existence of personality disorders (p = 0.015). 

## Discussion

The present study showed that somatization, hysteria, and pollyannaish were the most common personality disorders among the studied nurses. History of recent unpleasant event in the past year was the only effective factor in existence of personality disorders in the studied nurses. 

**Table 1:** Baseline characteristics of the questioned participants

**Variables**	**Number (%)**
**Marital status**	
Married	54 (52.9)
Single	47 (46.1)
Divorced	1 (1)
**Employment status**	
Stable	61 (59.8)
Unstable	41 (40.2)
**Work shift **	
Day	7 (6.9)
Night	11 (10.8)
Variable	84 (82.4)
**Income (US Dollar/month)**	
<500	29 (28.4)
≥500	73 (71.4)
Sole breadwinner	
Yes	6 (5.9)
No	96 (94.1)
**Medical history**	
Yes	15 (14.7)
No	87 (85.3)
**Drug history**	
Yes	14 (13.7)
No	88 (86.3)
**Recent history of trauma **	
Yes	5 (4.9)
No	97 (95.1)
**Recent history of unpleasant event**	
Yes	34 (33.3)
No	68 (66.7)

**Table 2:** Frequency of detected personality disorders among studied emergency nurses based on MMPI-2 questionnaire

**Disorders**	**Number (%)**
**Somatization**	9 (8.8)
**Hysteria**	7 (6.9)
**Pollyannaish**	5 (4.9)
**Depression**	3 (2.9)
**Reactive Depression**	2 (2.0)
**Schizopora**	2 (2.0)
**Hypochondriasis**	2 (2.0)
**Shy**	1 (1.0)
**Anxiety**	1 (1.0)

**Table 3:** The relationship of baseline characteristics of the studied population with personality disorders

**Variable **	Personality disorders		**P**
	**Absent**	**Present**	
**Age (year)**	30.1 ± 5.6	30.4 ± 5.7	0.808
**Mean working (hours/month)**	212.8 ± 51.8	206.6 ± 38.4	0.122
**Mean working experience (years)**	4.0 ± 3.4	4.3 ± 4.1	0.234
**Marital status**			
Single	31 (44.3)	16 (50)	0.709
Married	38 (54.3)	16 (50)	
Divorced	1 (1.4)	0 (0)	
**Employment status**			
Unstable	10 (19.6)	5 (20)	0.597
Stable	41 (80.4)	20 (80)	
**Working shift type**			
Day	4 (5.7)	3 (9.4)	0.720
Night	7 (10)	4 (12.5)	
Variable	59 (84.3)	25 (78.1)	
**Income (US Dollars/month)**			
< 500	19 (27.1)	10 (31.3)	0.420
≥ 500	51 (72.9)	22 (68.8)	
Sole breadwinner			
No	64 (91.4)	32 (100)	0.097
Yes	6 (8.6)	0 (0)	
**Medical history**			
No	61 (87.1)	26 (81.3)	0.309
Yes	9 (12.9)	6 (18.8)	
**Drug history**			
No	62 (88.6)	26 (81.3)	0.242
Yes	8 (11.4)	6 (18.8)	
**Recent history of unpleasant event**			
No	52 (74.3)	16 (50)	0.015
Yes	18 (25.7)	16 (50)	
**Recent history of trauma**			
No	68 (97.1)	29 (90.6)	0.176
Yes	2 (2.9)	3 (9.4)	

Data were presented as mean ± standard deviation or number and percentage.

The prevalence of personality disorders in the general population has been reported to be 4.4 – 10.6% (12-14). However, the rate is much higher among the participants of this study (31.4%). It should not be overlooked that some personality disorders remain hidden and symptoms only show when the individual is under workplace stress. 

Mealer et al. reported 18% more anxiety and 11% higher depression rates in intensive care unit (ICU) nurses compared to the general population (29% more in total) (15). In the present study, prevalence of anxiety and depression were 1% and 4.9%, respectively. In this study, nurses were selected from emergency departments. Exposure of the nurses to death scenes and dying patients for a long time can take a toll on their mental wellbeing (15). This is backed up by the findings of a study that showed emotional responses and psychophysiologic outcomes are more severe in nurses who have witnessed death and serious injuries in comparison with others and therefore, individuals in this group are more prone to post-traumatic stress disorders (16). 

In contrast, Escribà-Agüir et al. showed that there is no evidence that workplace and workload negatively impact presentation of burnout syndrome (2). However, Yong et al. measured saliva cortisol and expressed that ED nurses have higher stress levels compared to nurses from other departments. They concluded that ED nurses are under more stress but this does not increase the risk of having mental diseases (17). In other words, ED nurses’ ability to adapt to stress might have prevented them from developing personality disorders and showing symptoms. Psychological resilience is a factor affecting mental disorders (15). Presence of psychological resilience results in a significant decrease in the prevalence of post-traumatic stress disorder, burnout syndrome and symptoms of depression and anxiety. Therefore, resilience is a defense mechanism that can increase the ability of the nurses and other medical staff to adapt to workplace stresses. Since this mechanism is acquisitive, training programs to upgrade the skills of the treatment staff regarding psychological resilience can decrease the symptoms of mental and personality disorders, and increase job satisfaction (15).

Environmental stressors are among the factors leading to mental and personality disorders (18, 19). The findings of this study also showed that history of recent unpleasant event in the past year is an independent factor that exerts an effect on the existence of personality disorders in nurses. Nevertheless, to reach a conclusion in this regard, more studies with proper design are needed.

One of the limitations of this study was its low sample size, which may influence the power of the study. The nature of evaluating personality disorders is another limitation of this study. In most cases, personality disorders are not a single problem and several diagnoses are made for an individual. Therefore, it is possible that the reported percentages are different from reality to some extent. In addition, the psychologist who makes the diagnosis also plays a role.

## Conclusion:

The present study showed that somatization, hysteria, and pollyannaish were the most common personality disorders among the emergency nurses. History of a recent unpleasant event in the past year was the only effective factor in existence of personality disorder in the studied nurses. 
